# The Canine Leishmaniosis Proteinogram: A Reference Serum Electrophoretic Atlas of 5544 Dogs over 18 Years

**DOI:** 10.3390/pathogens15101045

**Published:** 2026-10-02

**Authors:** Ricardo Lopes, Alessia Giordano, Cristina Costa Santos, Hugo Lima de Carvalho, Filipe Sampaio, Cátia Fernandes, Ana Patrícia Lopes, Elsa Leclerc Duarte, Ana Cláudia Coelho, Luís Cardoso

**Affiliations:** 1 CEDIVET Veterinary Laboratories, Lionesa Business Hub, R. Lionesa 446 C24, 4465-671 Leça do Balio, Portugal; lopes.rmv@gmail.com (R.L.); hugo.carvalho@cedivet.pt (H.L.d.C.); filipe.sampaio@cedivet.pt (F.S.); 2 Department of Veterinary Sciences, University of Trás-os-Montes e Alto Douro (UTAD), 5000-801 Vila Real, Portugal; aplopes@utad.pt (A.P.L.); accoelho@utad.pt (A.C.C.); 3 Department of Veterinary and Animal Sciences, CESPU University, 4585-116 Gandra, Portugal; 4 UNIPRO—Oral Pathology and Rehabilitation Research Unit, CESPU University, 4585-116 Gandra, Portugal; 5 Department of Veterinary Medicine and Animal Sciences, University of Milan, 26900 Lodi, Italy; alessia.giordano@unimi.it; 6 RISE-Health, Department of Community Medicine, Information and Health Decision Sciences, Faculty of Medicine, University of Porto, Alameda Prof. Hernâni Monteiro, 4200-319 Porto, Portugal; csantos@med.up.pt; 7 Cytology and Hematology Diagnostic Services, Laboratory of Histology and Embryology, Department of Microscopy, ICBAS-School of Medicine and Biomedical Sciences, University of Porto (U. Porto), Rua de Jorge Viterbo Ferreira, 228, 4050-313 Porto, Portugal; 8 AniCura Santa Marinha Veterinary Hospital, R. Dom Henrique de Cernache 183, 4400-625 Vila Nova de Gaia, Portugal; catia.fernandes@anicura.pt; 9 Animal and Veterinary Research Centre (CECAV), Associate Laboratory for Animal and Veterinary Sciences (AL4AnimalS), University of Trás-os-Montes e Alto Douro (UTAD), 5000-801 Vila Real, Portugal; 10 Department of Veterinary Medicine, School of Science and Technology, University of Évora, Polo da Mitra, Apartado 94, 7002-554 Évora, Portugal; emld@uevora.pt; 11 Mediterranean Institute for Agriculture, Environment and Development (MED), Global Change and Sustainability Institute (CHANGE), University of Évora, Polo da Mitra, Apartado 94, 7002-554 Évora, Portugal

**Keywords:** canine leishmaniosis, capillary electrophoresis, clinical pathology, dysproteinaemia, gamma globulins, *Leishmania infantum*, proteinogram, serological intensity, serum protein electrophoresis

## Abstract

Serum protein capillary electrophoresis can characterise dysproteinaemia in dogs tested for canine leishmaniosis (CanL) as an integrated clinicopathological phenotype of humoral immune activation. This retrospective laboratory-based study evaluated serum protein electrophoretic patterns in 5544 dogs tested for CanL in Portugal between 2009 and 2026. Anti-*Leishmania* antibody results were classified into five ordered categories: negative, inconclusive, low positive, positive/high positive and very high positive. Total protein, albumin, alpha-1, alpha-2, beta and gamma fractions, albumin-to-globulin and albumin-to-gamma-globulin ratios were analysed. A separately defined reference subpopulation of 254 clinically healthy seronegative dogs aged ≥ 1 to <4 years was used to establish study-specific non-parametric 95% reference intervals. After excluding inconclusive results, binary analysis included 2454 seronegative and 2910 seropositive dogs. Seropositive dogs had higher median gamma globulins than seronegative dogs (1.5 vs. 0.8 g/dL; *p* < 0.001), with lower albumin-to-globulin (0.7 vs. 1.0; *p* < 0.001) and albumin-to-gamma-globulin ratios (1.9 vs. 4.2; *p* < 0.001). Across the ordinal serological scale, gamma globulins increased from 0.8 to 2.1 g/dL (*p* < 0.001), whereas the albumin-to-gamma-globulin ratio decreased from 4.2 to 1.2. Gamma globulins showed the strongest ordinal association (*ρ* = 0.522; *p* < 0.001) and moderate single-marker discrimination of seropositivity (area under the receiver operating characteristic curve = 0.768; 95% confidence interval: 0.755–0.780). These findings define the CanL proteinogram as a progressive, quantifiable immunopathological phenotype, dominated by gamma-region expansion and reciprocal albumin decline, supporting serum protein electrophoresis as a reference tool for interpretation and monitoring.

## 1. Introduction

Serum protein electrophoresis is one of the few routine laboratory techniques capable of transforming a simple biochemical abnormality into an interpretable representation of host immunobiology [1,2]. Rather than merely confirming hyperproteinaemia or hyperglobulinaemia, it resolves the serum protein compartment into a structured profile of albumin, alpha (α), beta (β) and gamma (γ) fractions, thereby providing insights into inflammation, acute-phase activation, antigenic stimulation, immunoglobulin production, protein loss and chronic tissue injury [3,4,5,6].

In dogs, serum protein electrophoresis has evolved from a largely descriptive technique for identifying dysproteinaemias into a clinically informative tool for recognising inflammatory, infectious, immune-mediated and neoplastic patterns [4,5,6]. Capillary zone electrophoresis is particularly valuable because of its automation, analytical speed and improved resolution compared with traditional electrophoretic methods [4,7]. However, electrophoretic interpretation remains platform-specific, and results should not be transferred uncritically between analytical systems or laboratories [4,5]. Absolute fraction concentrations are calculated from the electrophoretic percentage and the total serum protein concentration, making the technique both quantitative and morphologically informative [4,5]. Its clinical value, however, depends on interpretation of the complete electrophoretic profile rather than on isolated fraction values.

The biological meaning of each electrophoretic fraction is distinct but interconnected. Albumin reflects hepatic synthesis, oncotic status, negative acute-phase behaviour and protein loss. Alpha fractions include several acute-phase proteins and therefore frequently mirror early or active inflammation [8,9]. Beta fractions comprise a heterogeneous group of proteins, including transferrin, complement, lipoproteins and selected immunoglobulins, and may become particularly relevant in inflammatory, parasitic and immune-complex-associated conditions. In dogs with severe glomerular protein loss, relative prominence of the alpha-2 and beta regions may also occur because high-molecular-weight proteins such as alpha-2-macroglobulin and lipoproteins are comparatively retained. These changes should therefore be interpreted alongside absolute fraction concentrations and renal parameters. The gamma region is predominantly composed of immunoglobulins, particularly immunoglobulin G (IgG), and is therefore closely linked to chronic antigenic stimulation and humoral immune activation [3,4,5,6]. The proteinogram consequently provides a systems-level view of the inflammatory and immune response, integrating biological processes that are often assessed separately through biochemistry, inflammatory markers, serology and clinical staging [8,9,10,11,12,13].

**Figure 1:**
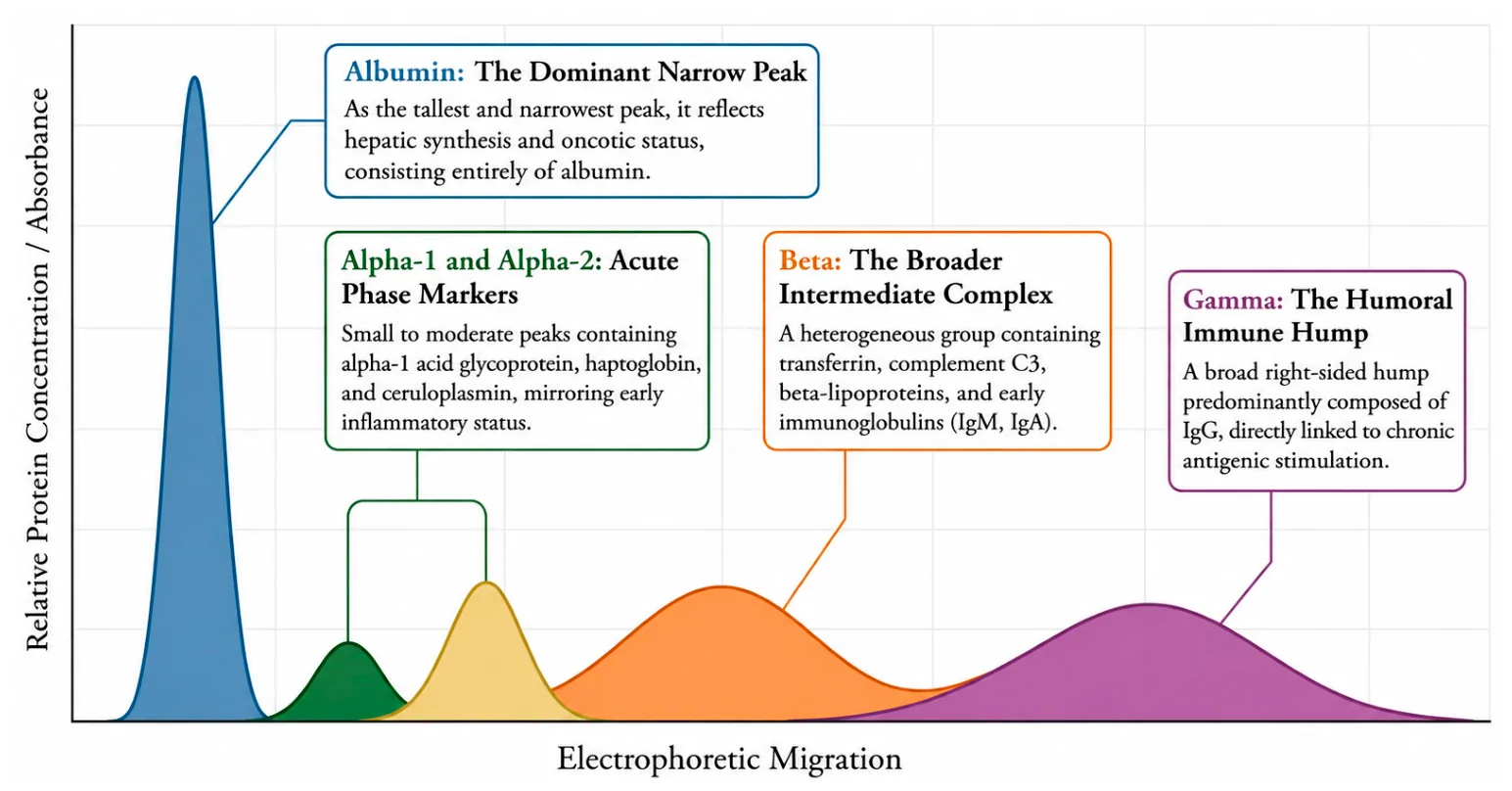
Schematic representation of the normal canine serum proteinogram. Original schematic created by the authors for this manuscript to support interpretation of the electrophoretic fractions. The electrophoretic trace illustrates the major serum protein fractions evaluated by capillary electrophoresis: albumin, α1-globulins, α2-globulins, β-globulins and γ-globulins. Albumin forms the dominant narrow peak and reflects hepatic synthesis, oncotic status and negative acute-phase behaviour. The α1 and α2 regions include several acute-phase proteins and are commonly associated with inflammatory activity. The β-region contains a heterogeneous group of proteins, including transferrin, complement components, β-lipoproteins and selected immunoglobulins. The γ region is dominated by immunoglobulins and reflects chronic antigenic stimulation and humoral immune activation. This schematic figure is intended to support interpretation of the electrophoretic patterns observed in dogs tested for leishmaniosis.

A schematic representation of the normal canine serum proteinogram is provided in Figure 1 to facilitate interpretation of the electrophoretic fractions discussed throughout this study.

Canine leishmaniosis (CanL) is caused predominantly by *Leishmania infantum* and represents one of the best naturally occurring disease models in veterinary medicine for investigating serum protein electrophoretic alterations associated with chronic inflammation and persistent immune stimulation [14,15]. CanL is a zoonotic vector-borne parasitosis with a wide clinical spectrum, which can range from a subclinical infection to a severe chronic systemic disease [16,17,18,19,20]. Dogs are the main domestic reservoir of *L. infantum*, and the disease expression depends strongly on the interaction between the parasite and the host immune response [16,21,22]. Protective cellular immunity may contribute to infection control, whereas exaggerated and persistent humoral activation is associated with progressive disease, circulating immune complexes, glomerular injury, systemic inflammation and characteristic dysproteinaemia [16,17,23,24]. The electrophoretic profile in CanL is not a passive laboratory abnormality; it rather constitutes a biochemical expression of the host immunopathological response.

The classical serum protein electrophoretic pattern associated with dogs clinically affected with leishmaniosis includes hypoalbuminaemia, a reduced albumin-to-globulin ratio, polyclonal hypergammaglobulinaemia and, in many dogs, variable beta-globulin expansion and acute-phase changes [16,17,23,25,26]. These findings are biologically consistent with chronic antigenic exposure, non-protective antibody production, immune-complex formation, renal protein loss and inflammatory redistribution of serum proteins [27,28]. None of these abnormalities is pathognomonic for leishmaniosis. Quantitative serology is not merely an exposure marker at all antibody levels: in a dog with compatible clinical or clinicopathological abnormalities, high anti-*Leishmania* antibody titres may confirm CanL, whereas low titres require further investigation [16,17,29,30]. Accordingly, SPE should not replace quantitative serology or parasite detection; its rationale is to localise dysproteinaemia to the albumin, acute-phase, β- or γ-regions and display broad, restricted or monoclonal-like morphology, thereby adding information unavailable from total protein or calculated globulin alone [3,4,5,6].

The relevance of serum protein electrophoresis in canine vector-borne diseases has recently gained renewed attention. In dogs naturally infected with *Dirofilaria immitis*, electrophoretic evaluation revealed frequent albumin and beta-globulin abnormalities, as well as treatment-associated improvement in albumin, beta fractions and the albumin-to-globulin ratio, supporting the role of the proteinogram as an adjunctive monitoring tool in parasitic disease [31]. More directly in CanL, a prospective treatment-monitoring study reported that dogs receiving meglumine antimoniate plus allopurinol showed short-term increases in albumin concentration and the albumin-to-globulin ratio, together with a decrease in gamma-globulins, supporting serum protein electrophoresis as a complementary tool for early assessment of therapeutic response [10]. In addition, a recent study evaluating serum protein electrophoresis and immunofixation in dogs seropositive for several vector-borne pathogens showed that most dysproteinaemias were inflammatory or polyclonal in nature, while only a small number of cases fulfilled combined electrophoretic and immunofixation criteria suggestive of a possible M-protein [32]. These observations emphasise two important principles: first, some vector-borne infections can induce characteristic serum protein electrophoretic patterns that reflect the host’s immunopathological response, and second, interpretation of broad, restricted or monoclonal-like bands requires both biological context and analytical caution [6,32,33].

In CanL, however, the proteinogram has often been treated as a descriptive accompaniment to diagnosis rather than as a primary object of investigation. Many studies report hypergammaglobulinaemia, hypoalbuminaemia and a decreased albumin-to-globulin ratio, but relatively few have examined whether the electrophoretic phenotype changes quantitatively across increasing anti-*Leishmania* serological intensity [14]. This distinction is critical. A binary comparison between seronegative and seropositive dogs may confirm that dysproteinaemia is common in infection, but it does not determine whether the serum protein profile is progressively reshaped as the humoral response intensifies.

A previous large-scale retrospective study in Portugal demonstrated that gamma-globulin concentrations and the albumin-to-globulin ratio were among the clinicopathological variables most clearly associated with anti-*Leishmania* enzyme-linked immunosorbent assay (ELISA) categories in dogs tested for *Leishmania* infection [14]. That study provided an important epidemiological and clinicopathological overview, showing that the protein profile was closely linked to serological evidence of infection. However, it was not designed to provide an in-depth, proteinogram-centred analysis of the electrophoretic phenotype. The extent to which the complete electrophoretic profile changes across increasing anti-*Leishmania* serological categories remains insufficiently characterised.

The present study was designed to characterise the quantitative relationship between the magnitude of anti-*Leishmania* seroreactivity and the serum protein electrophoretic phenotype in a large cohort of dogs tested for CanL. We hypothesised that increasing serological categories would be associated with progressive gamma-globulin expansion, decreasing albumin concentrations and relative proportions, and declining albumin-to-globulin and albumin-to-gamma-globulin ratios. Because the electrophoretic profile may also be influenced by renal protein loss, hepatic dysfunction and systemic inflammation, it was interpreted as an integrated clinicopathological phenotype rather than solely as a direct measure of humoral immune activation. Compared with the previous Portuguese study [14], the present work provides a proteinogram-focused analysis combining five ordered ELISA categories, ordinal effect sizes, exploratory receiver operating characteristic (ROC) analysis, adjusted regression, representative electropherograms and formal study-specific reference intervals. It thereby provides a robust quantitative framework for interpreting proteinogram alterations associated with increasing anti-*Leishmania* serological intensity rather than clinically staged disease severity. In this manuscript, “CanL proteinogram” denotes the electrophoretic phenotype examined within the diagnostic and pathobiological context of CanL, it does not imply that every seroreactive dog had clinically staged disease. Likewise, “reference atlas” denotes a comparative interpretive benchmark of quantitative distributions and representative traces, together with de novo reference intervals derived from the healthy subpopulation. A secondary objective was to establish formal study-specific reference intervals in a separately defined population of clinically healthy, anti-*Leishmania*-seronegative young adult dogs.

## 2. Materials and Methods

### 2.1. Study Design and Setting

This retrospective, laboratory-based, analytical study used records from domestic dogs submitted for *L. infantum* serology and serum protein electrophoresis between 2009 and 2026. Samples were submitted to CEDIVET Veterinary Laboratories (Porto, Portugal) by veterinary medical centres located in mainland Portugal and the autonomous insular regions.

The study was conceived as a proteinogram-centred extension of previous work evaluating epidemiological and clinicopathological relationships in CanL. Whereas the previous study provided a broad epidemiological and clinicopathological assessment in dogs tested in Portugal, the present study was specifically designed to evaluate the serum protein electrophoretic phenotype across increasing serological categories. This dataset is independent of the previously published cohort [14], with no record-level overlap, although cross-laboratory attendance by the same dog cannot be excluded.

During the study period, samples were received from 587 veterinary medical centres, including clinics and hospitals. Along with the biological sample, a laboratory request form was submitted by the referring veterinarian and included, when available, information on breed, sex, age, date of sampling, clinical suspicion or epidemiological indication for testing, and the requested laboratory analyses.

The study population should therefore be interpreted as a laboratory-tested canine population rather than as a population-based prevalence sample. Dogs were tested because of clinical suspicion of CanL, residence in or exposure to endemic areas, contact with potentially infected animals, previous or recent travel to areas where CanL occurs, or other clinical or epidemiological reasons justifying *Leishmania* testing.

Serological status was used as the main grouping variable. The study was reported in accordance with relevant items of the STROBE statement for observational studies. Throughout, “seronegative”, “seropositive” and “serological intensity” refer strictly to ELISA categories and do not retrospectively classify every dog as infected or clinically diseased. Therefore, the analyses should be interpreted as associations between anti-*Leishmania* serological intensity and the serum protein electrophoretic phenotype, rather than as estimates of clinical disease prevalence or diagnostic accuracy for CanL.

### 2.2. Eligibility Criteria and Study Population

Dogs were eligible for inclusion if they had available results for anti-*Leishmania* serology and serum protein electrophoresis. Only domestic dogs (*Canis lupus familiaris*) were included. Records were reviewed before analysis to identify repeated submissions. When repeated submissions from the same animal were detected, only the first chronological record with complete anti-*Leishmania* serology and serum protein electrophoresis results was retained. The final working database included 5544 dogs without repeated identifiers.

Dogs with inconclusive serological results were retained as a separate category in analyses using the complete ordinal serological scale but were excluded from binary comparisons between seronegative and seropositive dogs. Dogs with missing age were retained in the main electrophoretic analyses but excluded from analyses requiring age as a variable.

### 2.3. Sample Processing and Serum Protein Electrophoresis

All biological samples were assessed for analytical suitability before processing. Samples with macroscopic or technical characteristics likely to compromise laboratory interpretation, including marked haemolysis, lipaemia, inadequate sample volume, inappropriate sample type or other relevant pre-analytical limitations, were not considered suitable for the corresponding analysis.

Serum total protein (TP) concentration was measured using the Beckman Coulter AU680e Automated Chemistry Analyzer^®^ (Beckman Coulter, Cassina De’Pecchi, Italy). Serum protein electrophoretic separation was performed by capillary electrophoresis using the Sebia^®^ Minicap Flex Piercing Analyser (Sebia, Lisses, France), according to the manufacturer’s instructions.

The electrophoretic profile included albumin, alpha-1, alpha-2, beta and gamma fractions. For each electrophoretic fraction, both the relative percentage and the absolute concentration were recorded. Absolute fraction concentrations were derived from the electrophoretic percentage and the total serum protein concentration.

The following variables were analysed: total protein, albumin percentage, albumin concentration, alpha-1 percentage, alpha-1 concentration, alpha-2 percentage, alpha-2 concentration, beta percentage, beta concentration, gamma percentage and gamma concentration. Two derived ratios were also analysed: the albumin-to-globulin ratio and the albumin-to-gamma-globulin ratio. The albumin-to-globulin ratio was calculated as albumin concentration divided by calculated globulin concentration. The albumin-to-gamma-globulin ratio was calculated as albumin concentration divided by gamma-globulin concentration.

### 2.4. Serological Testing and Classification

Anti-*Leishmania* antibodies were detected using a commercial indirect ELISA based on *L. infantum* antigens, commercially available during the study period as LEISCAN^®^ *Leishmania* ELISA Test/CIVTEST^®^ Canis *Leishmania* (HIPRA, Amer, Girona, Spain), according to the manufacturer’s instructions. Published independent validation of the LEISCAN^®^ assay reported a sensitivity of 92.5% and a specificity of 100% [34].

Results were expressed as ELISA ratio (Rz) values and interpreted according to the manufacturer’s cut-off system: negative, Rz ≤ 0.9; inconclusive, 0.9 < Rz ≤ 1.1; low positive, 1.1 < Rz ≤ 1.5; positive to high positive, 1.5 < Rz ≤ 3; and very high positive, Rz > 3.

For binary analyses, dogs were classified as seronegative when Rz ≤ 0.9 and seropositive when Rz > 1.1. Dogs with inconclusive results were excluded from binary seronegative versus seropositive comparisons.

### 2.5. Data Management and Quality Control

All available data were extracted from the laboratory information system and organised in Microsoft Excel^®^ (Microsoft Corporation, Redmond, WA, USA) before statistical analysis. Records were extracted from the laboratory information system, Sislab^®^ (Glintt, Global Intelligent Technologies, Lisbon, Portugal), in which samples are automatically assigned unique identification codes by the laboratory server and software. The database was then checked for duplicate records, missing values, coding inconsistencies, and biologically implausible values.

The internal coherence of the proteinogram was also reviewed before analysis. Because absolute electrophoretic fraction concentrations were derived from total serum protein concentration and electrophoretic fraction percentage, the relationships between total protein, relative fractions and absolute fraction values were evaluated during data cleaning. The presence of extreme outliers was carefully evaluated, and values were retained only when no pre-analytical, analytical or data-entry issue was identified and the internal relationships between total protein, relative fractions and absolute fraction concentrations remained coherent.

### 2.6. Selection and Preparation of Representative Electropherograms

Representative serum protein electropherograms were selected to illustrate the main electrophoretic patterns observed across anti-*Leishmania* serological categories and relevant dysproteinaemic phenotypes. Selection was based on the complete quantitative electrophoretic profile, including total protein concentration, albumin, α1-, α2-, β- and γ-globulin percentages and concentrations, and the albumin-to-globulin and albumin-to-γ-globulin ratios. Although all fractions were considered during selection, the atlas was organised around the most visually distinctive β- and γ-region phenotypes.

For the serological progression figure, representative electropherograms were selected from dogs classified as negative, inconclusive, low positive, positive/high positive and very high positive. For the atlas figure, additional electropherograms were selected to illustrate near-normal profiles, mild γ-globulin expansion, β-region expansion, β–γ expansion, severe dysproteinaemia and marked hypergammaglobulinaemia.

Electropherogram images were extracted from original laboratory reports and edited only to remove identifying information. No modification was made to the electrophoretic trace itself. The representative images were used for visual illustration only and were not included as independent observations in the statistical analyses. Because electropherogram display scaling may vary between reports, visual comparison of peak morphology should be interpreted together with the corresponding quantitative fraction values.

### 2.7. Statistical Analysis

Data were extracted from Sislab^®^ (Glintt, Global Intelligent Technologies, Lisbon, Portugal), organised in Microsoft Excel^®^ (Microsoft Corporation, Redmond, WA, USA) and checked for duplicate records, missing data, implausible values and coding inconsistencies before analysis. All statistical procedures were performed using JMP^®^ version 18.0.0 (SAS Institute Inc., Cary, NC, USA) and DATAtab^®^ (numiqo e.U., Graz, Austria, 2026).

Descriptive statistics were calculated for demographic, serological and electrophoretic variables. Continuous variables were summarised as median, interquartile range (IQR), minimum and maximum because most variables were non-normally distributed and clinically skewed. Categorical variables were summarised as absolute and relative frequencies.

For binary analyses, inconclusive serological results were excluded, and seronegative and seropositive dogs were compared. The association between sex and serological status was assessed using the chi-square test, with effect size expressed as Cramer’s *V*. Age and electrophoretic variables were compared between seronegative and seropositive dogs using the Mann–Whitney U test, with effect size reported as *r*.

For ordinal analyses, the complete five-category serological scale was used: negative, inconclusive, low positive, positive to high positive and very high positive. Differences in electrophoretic variables across serological categories were assessed using the Kruskal–Wallis test, with omnibus effect size expressed as epsilon-squared (ε^2^). When appropriate, post hoc pairwise comparisons were performed using Dunn’s tests with Bonferroni adjustment. Ordinal associations between serological category and electrophoretic variables were assessed using Spearman’s rank correlation coefficient (*ρ*). The primary ordinal electrophoretic outcome was γ-globulin concentration, selected a priori because it most directly reflects humoral immune activation in CanL.

ROC curve analysis was used to evaluate the single-marker discriminatory performance of selected electrophoretic variables for differentiating seropositive from seronegative dogs. The area under the curve (AUC) was reported with 95% confidence intervals (CIs) to assess discriminatory performance of each electrophoretic variable. For the best-performing marker, exploratory cut-off values were identified using the Youden index (J), and operating characteristics relative to binary ELISA status were described using sensitivity, specificity, and positive (LR+) and negative (LR−) likelihood ratios.

To assess whether the association between the electrophoretic variable showing the highest discriminatory performance in ROC analysis and serological status was independent of basic demographic and temporal covariates, binary logistic regression was performed with seropositive status as the dependent variable and the selected electrophoretic variable, age, sex and year of sampling as explanatory variables. Regression results were reported as adjusted odds ratios (OR) with 95% CI. As an exploratory analysis, ordinal logistic regression was performed using the five-category serological scale as the dependent variable and the same explanatory variables to evaluate whether the association remained consistent across increasing levels of serological intensity. Adjusted OR with 95% CI were reported.

All tests were two-sided, and statistical significance was set at *p* < 0.05 [35]. However, interpretation prioritised effect size, CI, consistency across analyses and biological plausibility rather than *p*-values alone.

### 2.8. Reference Subpopulation and Reference-Interval Estimation

A reference subpopulation was defined before interval calculation from unique dogs submitted for pre-vaccination anti-*Leishmania* testing. Dogs were eligible when the referring veterinarian identified them as clinically healthy, recorded no history or clinical evidence of disease, the ELISA result was negative (Rz ≤ 0.9) and age at sampling was ≥1 and <4 years. Age was treated as a continuous variable. Normal haematological, biochemical or urinalysis results were not inferred.

In accordance with American Society for Veterinary Clinical Pathology (ASVCP) and Clinical and Laboratory Standards Institute (CLSI) recommendations [36,37], central 95% reference intervals were estimated non-parametrically from the 2.5th and 97.5th percentiles, with 90% CI. Potential outliers were screened using Tukey and Dixon–Reed methods and were not excluded solely on statistical grounds. Potential partitioning by sex, age and sampling period was assessed using non-parametric analyses with Benjamini–Hochberg adjustment.

## 3. Results

### 3.1. Study Population and Serological Classification

The final dataset included 5544 dogs tested for anti-*Leishmania* antibodies and serum protein electrophoresis. Of these, 2454 dogs were classified as seronegative, 180 as inconclusive, 368 as low positive, 1229 as positive to high positive and 1313 as very high positive. After exclusion of inconclusive results, the binary analysis included 5364 dogs, comprising 2454 seronegative dogs and 2910 seropositive dogs.

Sex was recorded for all dogs included in the binary dataset, which comprised 2074 females and 3290 males. Among females, 989 were seronegative and 1085 were seropositive. Among males, 1465 were seronegative and 1825 were seropositive. The proportion of seropositive dogs was 52.3% in females and 55.5% in males. Sex was statistically associated with serological status, although the magnitude of association was very small (χ^2^ = 5.11, *df* = 1, *p* = 0.024; Cramer’s *V* = 0.03).

From the 5364 dogs included in the binary serological analysis, age data were available for 4861 individuals. For the remaining 503 dogs (9.4%), age was not specified in the requisition file, and these were therefore excluded from age-related analyses. Among dogs with available age data, age ranged from 0.5 (6 months) to 22 years. Age group was associated with serological status (χ^2^ = 129.27, *df* = 4, *p* < 0.001; Cramer’s *V* = 0.16), with the highest seropositive proportion observed in dogs aged 2 to <6 years. In the continuous-age comparison, seropositive dogs were slightly younger than seronegative dogs (median age 6 years [IQR: 4–9] vs. 7 years [IQR: 4–10]; Mann–Whitney U = 2,816,360, *p* = 0.012), although the effect size was very small (*r* = 0.05). Table 1 displays the proportion of seropositive dogs according to age group.

### 3.2. Serum Protein Electrophoretic Profile in Seronegative and Seropositive Dogs

Compared with seronegative dogs, seropositive dogs had higher total protein, beta-globulin and gamma-globulin concentrations, lower albumin concentration and percentage, and lower albumin-to-globulin and albumin-to-gamma-globulin ratios (Table 2). All variables differed significantly between groups, except alpha-1-globulin concentration (*p* = 0.483) and beta-globulin percentage (*p* = 0.499). The difference in age had *p* = 0.012, whereas all other significant differences had *p* < 0.001. The largest effect sizes were observed for gamma-globulin concentration (*r* = 0.462), gamma-globulin percentage (*r* = 0.461), the albumin-to-gamma-globulin ratio (*r* = 0.453), albumin percentage (*r* = 0.363) and the albumin-to-globulin ratio (*r* = 0.363).

### 3.3. Serum Protein Electrophoretic Profile Across the Ordinal Serological Scale

When the complete five-category serological scale was analysed, gamma-globulin concentration increased progressively across categories. Median gamma-globulin concentration was 0.8 g/dL in seronegative dogs, 0.9 g/dL in inconclusive dogs, 1.0 g/dL in low-positive dogs, 1.3 g/dL in positive/high-positive dogs and 2.1 g/dL in very high-positive dogs. This ordinal gradient was statistically strong and biologically coherent (Kruskal–Wallis χ^2^ = 1553.91, *df* = 4, *p* < 0.001; ε^2^ = 0.280), indicating that increasing anti-*Leishmania* serological intensity was accompanied by a marked progressive expansion of the gamma-globulin fraction.

**Figure 2:**
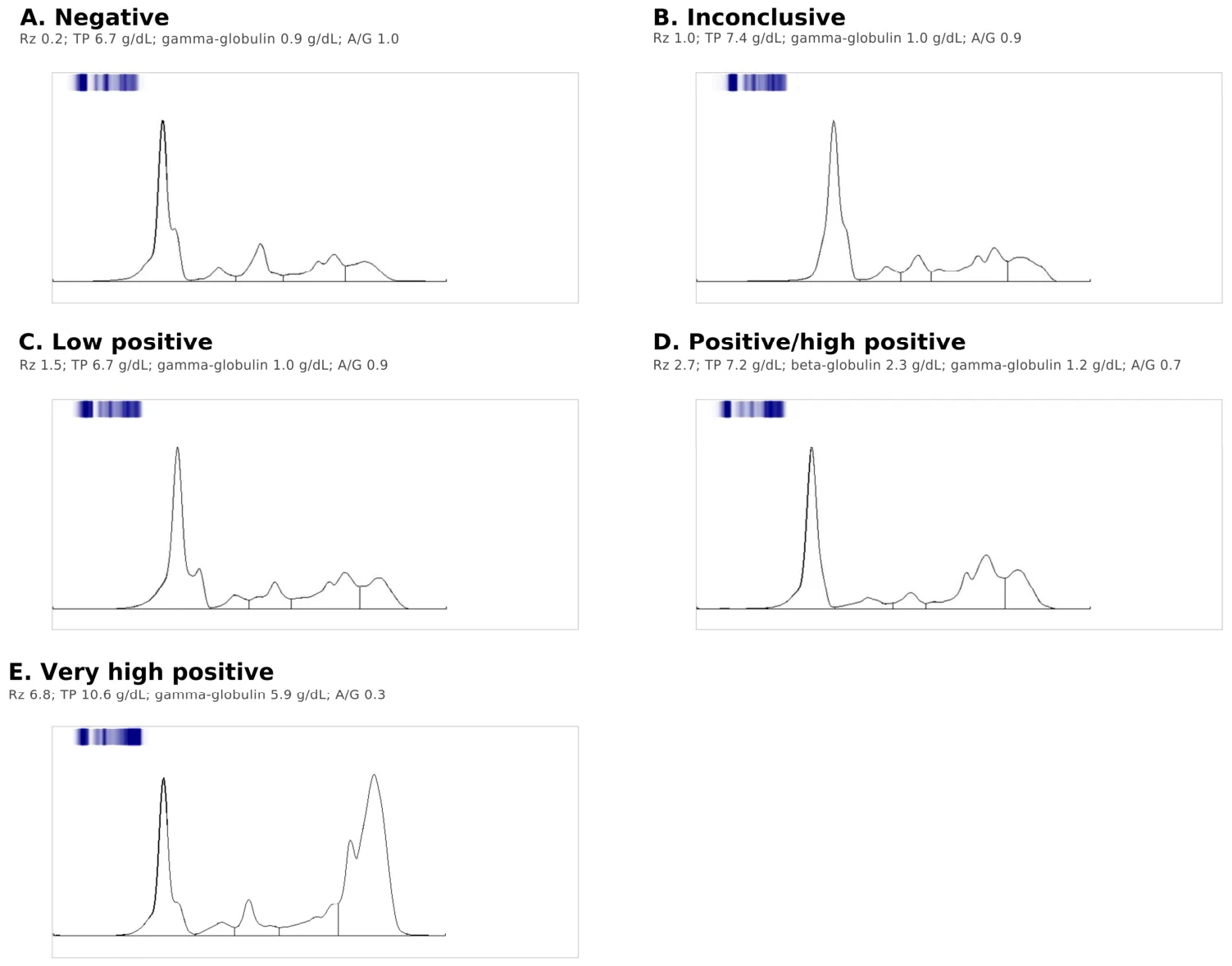
Representative serum protein capillary electrophoretic patterns across anti-*Leishmania* serological categories. Representative electropherograms from dogs classified as negative (**A**), inconclusive (**B**), low positive (**C**), positive/high positive (**D**) and very high positive (**E**) according to anti-*Leishmania* enzyme-linked immunosorbent assay (ELISA) results. The panels illustrate the transition from a near-normal electrophoretic profile to progressively more evident dysproteinaemia, mainly characterised by expansion of the γ-globulin region and reduction in albumin-related proportions. The traces are shown as representative examples and should be interpreted together with the corresponding quantitative electrophoretic values. Rz, ELISA ratio; TP, total protein; ALB, albumin; A/G, albumin-to-globulin ratio.

The albumin-to-gamma-globulin ratio declined in parallel across the same categories, with medians of 4.18 (IQR: 2.61–6.10), 3.67 (2.61–5.25), 3.50 (1.99–5.30), 2.36 (1.20–3.85) and 1.22 (0.69–2.21) in negative, inconclusive, low-positive, positive/high-positive and very high-positive dogs, respectively (Kruskal–Wallis χ^2^ = 1508.60, *df* = 4, *p* < 0.001; ε^2^ = 0.272). Representative electropherograms illustrating this progressive serological gradient are shown in Figure 2.

Bonferroni-adjusted Dunn’s post hoc comparisons showed that negative and inconclusive dogs did not differ significantly, and that inconclusive and low-positive dogs also did not differ significantly after adjustment. All other pairwise comparisons were statistically significant. Table 3 summarises gamma-globulin concentration, 95% CI and post hoc groupings according to anti-*Leishmania* serological category.

As shown in Table 4, the strongest ordinal associations with increasing anti-*Leishmania* serological category were observed for gamma-globulin concentration (*ρ* = 0.522), γ-globulin percentage (*ρ* = 0.521), albumin-to-gamma-globulin ratio (*ρ* = −0.513), albumin percentage (*ρ* = −0.417) and albumin-to-globulin ratio (*ρ* = −0.416). In contrast, beta-globulin concentration showed only a weak ordinal association (*ρ* = 0.134), and alpha-1-globulin concentration showed no meaningful ordinal association. This pattern indicates that the serological gradient was dominated by progressive γ-region expansion and reciprocal albumin-related decline, rather than by a uniform increase in all globulin fractions. Gamma-globulin concentration showed the strongest ordinal association with increasing anti-*Leishmania* serological category (*ρ* = 0.522; *p* < 0.001).

### 3.4. Discriminatory Performance and Adjusted Modelling

**Figure 3:**
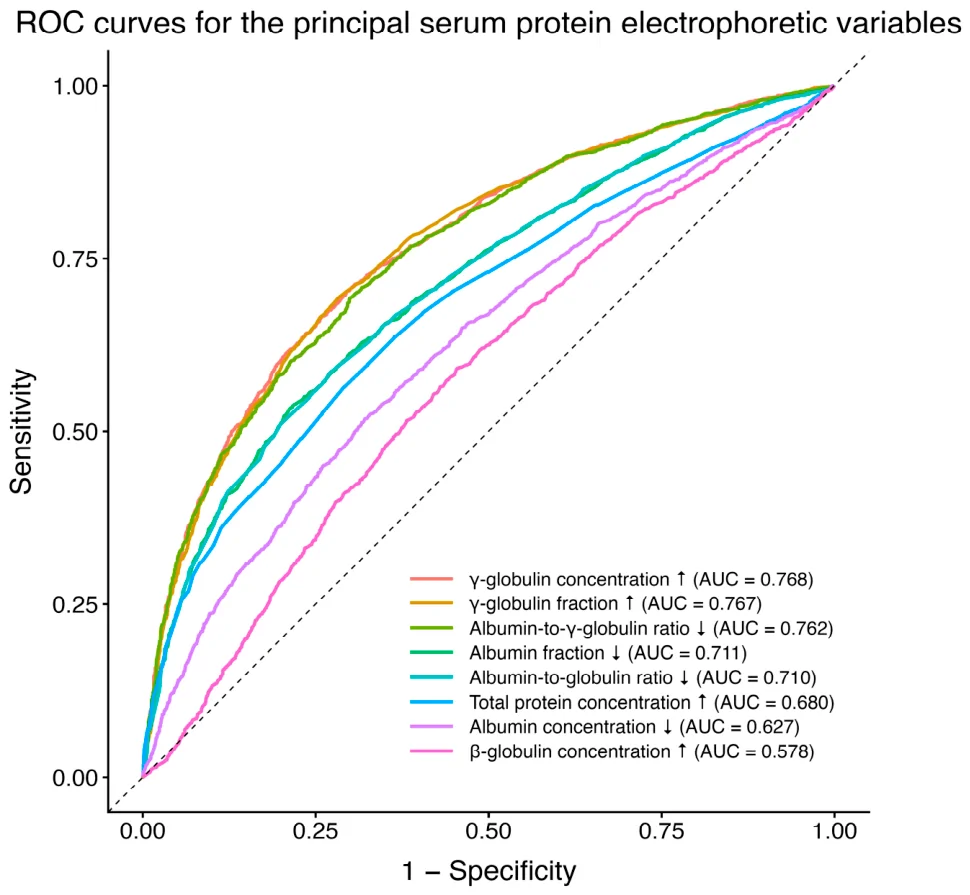
Receiver operating characteristic (ROC) curves for the principal serum protein electrophoretic variables in discriminating seropositive from seronegative dogs. γ-globulin concentration, γ-globulin fraction, total protein concentration, and β-globulin concentration increased with seropositivity, whereas albumin-to-γ-globulin ratio, albumin fraction, albumin-to-globulin ratio, and albumin concentration decreased. Curves are displayed with the orientation that maximises the area under the curve (AUC), allowing direct comparison of their discriminatory performance relative to binary serological status.

In single-marker ROC analysis, gamma-globulin concentration showed the highest, but moderate, discriminatory performance for differentiating seropositive from seronegative dogs, with an AUC of 0.768 (95% CI: 0.755–0.780) as shown in Figure 3. Using the Youden index, an exploratory and dataset-specific gamma-globulin cut-off of approximately 1.05 g/dL yielded a sensitivity of 70.2% and a specificity of 70.5% (LR+ = 2.38; LR− = 0.42). Table 5 summarises the AUC and 95% CI for all electrophoretic variables evaluated. The 1.05 g/dL value therefore describes separation of binary ELISA categories within this dataset and is not a diagnostic cut-off for CanL.

Overall, the electrophoretic variables most closely associated with anti-*Leishmania* serological positivity were gamma-globulin concentration, gamma-globulin percentage, the albumin-to-gamma-globulin ratio and the albumin-to-globulin ratio. This pattern supports the interpretation of the proteinogram as an integrated electrophoretic phenotype of humoral immune activation in dogs tested for CanL.

**Figure 4:**
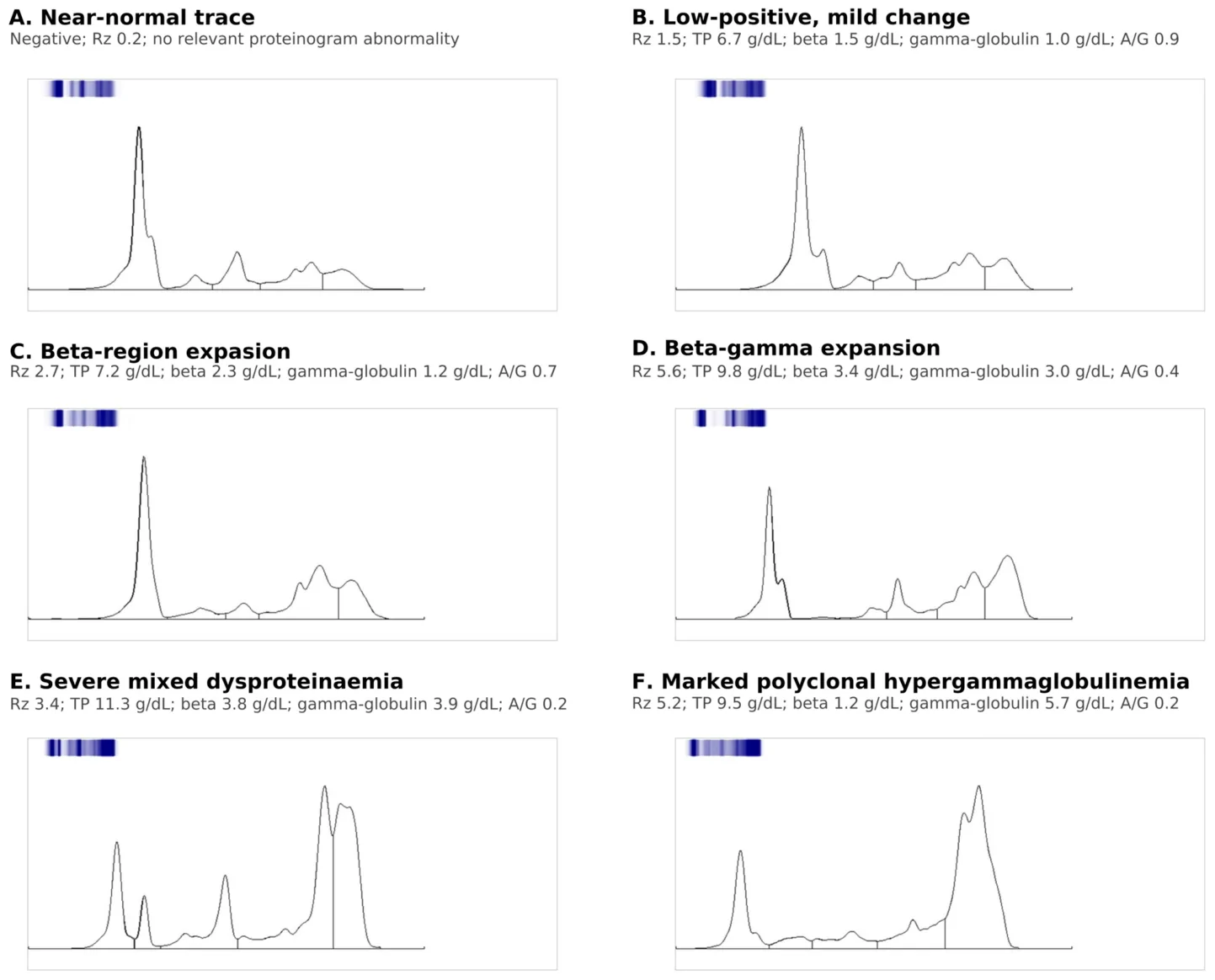
Atlas of representative serum protein capillary electrophoretic patterns in dogs tested for leishmaniosis. Representative electropherograms were selected to illustrate the morphological spectrum of serum protein electrophoretic patterns associated with different anti-*Leishmania* serological intensities. (**A**) Near-normal electrophoretic trace from a seronegative dog, with no relevant proteinogram abnormality and preserved albumin-to-globulin balance. (**B**) Low-positive anti-*Leishmania* serology with mild electrophoretic change, characterised by a slight γ-globulin increase and a preserved albumin-to-globulin ratio. (**C**) β-region expansion in a positive/high-positive dog, showing predominant β-globulin increase with only mild γ-globulin involvement. (**D**) β–γ expansion in a very high-positive dog, characterised by concurrent enlargement of the β- and γ-globulin regions. (**E**) Severe mixed dysproteinaemia in a very high-positive dog, with marked hypoalbuminaemia, β-globulin expansion, γ-globulin expansion and a severely reduced albumin-to-globulin ratio. (**F**) Marked γ-globulin dominance in a very high-positive dog, showing pronounced γ-region expansion. These traces are intended as representative visual examples and should be interpreted together with the corresponding quantitative fraction values.

Representative electropherograms were also selected to illustrate the main electrophoretic phenotypes observed in dogs tested for CanL (Figure 4). These included near-normal profiles, mild gamma-globulin expansion, β-region expansion, β–γ expansion, severe dysproteinaemia and marked hypergammaglobulinaemia. These examples are intended to support visual interpretation of the proteinogram and should be regarded as illustrative morphologic patterns rather than independent statistical observations.

The association between gamma-globulin concentration and serological status remained strong after adjustment for age, sex and year of sampling. In binary logistic regression, each 1 g/dL increase in gamma-globulin concentration was associated with a 2.77-fold increase in the odds of seropositivity (adjusted OR = 2.77; 95% CI: 2.54–3.02; *p* < 0.001). Older age was associated with slightly lower odds of seropositivity, whereas male sex and more recent years of sampling were associated with slightly higher odds.

In an exploratory ordinal logistic regression model using the five-category serological scale, each 1 g/dL increase in gamma-globulin concentration was likewise associated with higher serological intensity after adjustment for age, sex, and year of sampling (adjusted OR = 2.28; 95% CI: 2.16–2.41; *p* < 0.001).

### 3.5. Reference Intervals in Clinically Healthy Seronegative Dogs

The reference subpopulation comprised 254 unique dogs (149 males and 105 females), all anti-*Leishmania* negative, aged ≥ 1 to <4 years and with complete electrophoretic data. The age strata contained 77 dogs aged ≥ 1 to <2 years, 95 aged ≥ 2 to <3 years and 82 aged ≥ 3 to <4 years. No observations were excluded during statistical analysis. The study-specific 95% reference intervals calculated from clinically healthy seronegative dogs are presented in Table 6 to support interpretation of the electrophoretic profiles.

The desirable ASVCP precision target was met for 22 of 26 reference-limit CIs. Females had modestly lower alpha-2-globulin percentage and concentration than males (Benjamini–Hochberg-adjusted *p* < 0.01), while age associations were weak (*ρ* = 0.18).

## 4. Discussion

This study provides a large-scale, proteinogram-centred analysis of how serum protein capillary electrophoretic patterns vary across increasing anti-*Leishmania* antibody titres, as reflected by the ordinal ELISA Rz categories, in dogs tested for CanL. The principal finding was a clear, graded expansion of the gamma-globulin region across the complete ordinal serological scale, accompanied by reciprocal reductions in albumin-related proportions and derived albumin-to-globulin and albumin-to-gamma-globulin ratios. This pattern was consistent in binary comparisons, ordinal analyses, effect-size estimates, ROC analysis and adjusted regression modelling, supporting the interpretation that the serum proteinogram captures a measurable electrophoretic phenotype of humoral immune activation in dogs tested for CanL. The principal novelty lies in combining the complete five-category serological scale, effect-size estimates, adjusted models and representative electropherograms in a proteinogram-centred cohort of this size. These findings describe an association with serological intensity and should not be interpreted as evidence of increasing clinical disease severity.

The most informative variable was gamma-globulin concentration. Its progressive increase from seronegative to very high-positive dogs indicates that the electrophoretic profile is not merely abnormal in seropositive animals, but changes quantitatively as the anti-*Leishmania* antibody response intensifies. This is biologically plausible, because CanL is characterised by chronic antigenic stimulation, exaggerated humoral immunity, immunoglobulin production and immune-complex-associated pathology [16,17,23]. Because IgG contributes predominantly to the γ-region and IgG-containing immune complexes may also contribute to γ-region expansion, the strong association between gamma-globulin concentration and increasing serological intensity is biologically expected [12,13,23]. Albumin concentration may decrease in dogs with CanL through several concurrent mechanisms, including reduced hepatic synthesis as part of the negative acute-phase response, renal loss associated with glomerular disease or protein-losing nephropathy, reduced nutritional intake in clinically affected animals and, in some cases, protein loss associated with severe cutaneous lesions [16,17,23,25,26]. The parallel decrease in albumin fraction, albumin-to-globulin ratio and albumin-to-gamma-globulin ratio further supports the view that the serology-associated proteinogram reflects a coordinated redistribution of serum proteins rather than an isolated increase in one fraction. Taken together, these findings support interpretation of the proteinogram as an integrated clinicopathological phenotype associated with anti-*Leishmania* serological intensity, rather than as a direct measure of humoral immunity alone.

These findings reinforce the classical description of CanL-associated dysproteinaemia, which includes hypoalbuminaemia, reduced albumin-to-globulin ratio, polyclonal hypergammaglobulinaemia and variable β-region involvement [16,17,23,25,29]. However, the present study extends that evidence in three important ways. First, it evaluates the proteinogram as the primary object of analysis rather than as an ancillary biochemical variable. Second, it uses a five-category serological scale, allowing the electrophoretic phenotype to be interpreted along a continuum of antibody burden. Third, the large sample size provides a stable quantitative framework for median values, CI, ordinal effect sizes and representative electrophoretic phenotypes.

The formal reference-interval component extends the atlas beyond descriptive distributions in the complete laboratory-tested cohort. The intervals were derived from a separately defined population of clinically healthy, seronegative young adult dogs and therefore provide a platform-specific baseline against which the progressive dysproteinaemia associated with increasing serological intensity can be interpreted. Their precision was generally acceptable, with 22 of 26 reference-limit CIs meeting the desirable ASVCP target.

The dominance of gamma-globulin-related variables is also clinically meaningful. In routine practice, total protein and calculated globulin values may suggest dysproteinaemia, but they cannot localise the protein shift or distinguish whether the change is driven mainly by albumin loss, acute-phase fractions, β-region expansion or γ-region immunoglobulin production. Capillary electrophoresis provides that localisation and transforms a non-specific biochemical abnormality into a more interpretable inflammatory and immunological profile [3,4,5,6]. In the present dataset, total protein was increased in seropositive dogs, but the strongest serological gradient was concentrated in the γ-region, indicating that the main electrophoretic signature of increasing serological intensity was humoral rather than a generalised increase in all fractions.

The beta fraction deserves a more cautious interpretation. Beta-globulin concentration was higher in seropositive dogs but showed only a weak ordinal association with increasing serological category. This suggests that β-region expansion may occur in a subset of dogs, possibly reflecting acute-phase proteins, complement, lipoproteins, selected immunoglobulins or beta-gamma transition phenomena, but it was not the dominant marker of increasing anti-*Leishmania* serological intensity. Beta-region abnormalities are non-specific and may also be influenced by inflammation, hepatic or renal disease, lipid metabolism, haemolysis, sample type and analytical method [3,4,5,6,38]. Therefore, beta expansion should be interpreted morphologically and quantitatively, and always in conjunction with the complete electrophoretic profile. Accordingly, β-region expansion should be regarded as a secondary, non-specific feature and should not be assigned interpretive weight equivalent to the γ-region/albumin pattern.

The representative electropherograms link quantitative data to recognisable patterns but are illustrative rather than diagnostic. They should be interpreted together with the corresponding fraction values and clinical context [1,10,13].

ROC curve results should be interpreted with restraint. Gamma-globulin concentration achieved the highest single-marker discriminatory performance for differentiating seropositive from seronegative dogs, but the AUC indicates moderate rather than definitive discrimination. Although the albumin-to-gamma-globulin ratio combines reciprocal changes in albumin and gamma-globulins, its AUC was slightly lower than that of γ-globulin concentration alone (0.762 versus 0.768). This may reflect additional variability in albumin arising from renal, hepatic, inflammatory and nutritional mechanisms unrelated directly to antibody production. The 1.05 g/dL value is an exploratory, dataset-specific threshold for separating ELISA categories and should not be interpreted as a diagnostic cut-off for CanL. This is especially important because serum protein electrophoresis is not specific for *Leishmania* infection, and similar dysproteinaemic patterns may occur in other chronic inflammatory, infectious, immune-mediated, hepatic, renal or neoplastic conditions [3,4,5,6,32]. Accordingly, the proteinogram should complement, not replace, quantitative serology, clinical assessment, parasite detection when indicated, urinalysis and renal evaluation [16,17,29].

The results also need to be interpreted within the broader context of canine vector-borne diseases. Analogous polyclonal or restricted γ-region expansion and reduced albumin-to-globulin ratios occur in dogs seroreactive to *Ehrlichia canis* or *Anaplasma phagocytophilum*, *Babesia gibsoni* or *Candidatus* Mycoplasma haematoparvum, and *D. immitis* [32]. Rare monoclonal hypergammaglobulinaemia with cryoglobulinaemia has also been reported in *L. infantum* infection and may interfere with automated analysers, reinforcing the need for careful sample handling and, where indicated, immunofixation [39].

Recent work combining serum protein electrophoresis and immunofixation in dogs seropositive for different vector-borne pathogens showed that most dysproteinaemias were polyclonal or restricted polyclonal in nature, whereas true M-protein-like patterns were uncommon [32]. This is directly relevant to CanL because intense or irregular immunoglobulin responses may occasionally produce restricted-looking peaks. Therefore, narrow or atypical bands in dogs with suspected or confirmed vector-borne infection should not be overinterpreted as neoplastic paraproteinaemia without appropriate clinical context and, when necessary, immunofixation or additional confirmatory testing.

Compared with previous Portuguese data, this study shifts the emphasis from broad clinicopathological association to electrophoretic phenotyping. The earlier 15-year retrospective study showed that gamma globulins and albumin-to-globulin ratio were among the laboratory variables most closely associated with anti-*Leishmania* ELISA results [14]. The present analysis confirms that observation in a larger proteinogram-focused cohort and demonstrates that the association is ordinal, quantitative and biologically coherent. In this sense, the current work should be presented as an extension rather than a repetition of previous findings: it reframes the proteinogram as an integrated clinicopathological readout and provides a more detailed atlas of the serum protein landscape associated with serological intensity.

### 4.1. Limitations

The study was retrospective and laboratory-based; therefore, the sample does not represent the general canine population and cannot be used to estimate disease prevalence. Dogs were submitted for clinical or epidemiological reasons, creating selection bias towards animals in which leishmaniosis testing was considered relevant. Clinical staging, treatment status, vaccination history, quantitative parasite detection, complete haematological and biochemical profiles, urinalysis, proteinuria, renal staging, acute-phase proteins and screening for co-infections were not uniformly available. These missing variables limit the ability to distinguish infection from active clinical disease, to assess renal or immune-complex contributions to hypoalbuminaemia, or to determine whether individual electrophoretic patterns reflect leishmaniosis alone or concurrent disease. This limitation prevents retrospective clinical classification of each dog. The recognised diagnostic weight of high quantitative serology applies only in a clinically compatible patient [16,25,30,34]. The large sample size improves precision within this laboratory-tested cohort but does not confer population representativeness. Residual confounding by renal or protein-losing disease, hepatic disease, systemic inflammation, co-infections, nutritional status, treatment, vaccination, clinical stage and other chronic diseases remains possible because only age, sex and year were included in the adjusted models. Accordingly, the models estimate associations conditional on those three covariates and do not establish causal or disease-specific effects. Detailed diagnoses and inflammatory status were unavailable for the remaining seronegative dogs, precluding reliable classification by infectious aetiology or inflammatory category.

Additional analytical considerations are also important. Capillary electrophoresis provides high-resolution, automated separation, but electrophoretic fraction values are method-specific and should not be transferred uncritically to other platforms or laboratories [1,3,4,5,6]. Absolute fraction concentrations were derived from total serum protein and relative electrophoretic percentages, which is standard practice but makes pre-analytical quality and total protein measurement central to interpretation. The representative electropherograms were selected for didactic illustration and were not analysed as independent observations. Although adjustment for age, sex and year supported the robustness of the gamma-globulin association, residual confounding cannot be excluded in a retrospective diagnostic dataset.

Finally, the reference subpopulation has additional limitations. Clinical health was defined from the referring veterinarian’s pre-vaccination assessment and available history; normal concurrent haematology, biochemistry and urinalysis were not mandatory inclusion criteria. The intervals are consequently restricted to anti-Leishmania-negative dogs aged ≥ 1 to <4 years analysed using the same system.

### 4.2. Future Recommendations

Future studies should validate these findings prospectively in clinically staged dogs, ideally integrating quantitative serology, parasite load, immunofixation, acute-phase proteins, renal and hepatic biomarkers, urinalysis, proteinuria, treatment response and long-term outcomes. Longitudinal designs would be particularly valuable to determine how rapidly albumin, beta and gamma fractions change after treatment, whether specific electrophoretic trajectories predict relapse or persistent renal damage, and whether combined indices such as the albumin-to-gamma-globulin ratio improve clinical monitoring beyond the conventional albumin-to-globulin ratio. Such work would determine whether the proteinogram can serve as a longitudinal marker of clinically staged disease activity. Prospective studies should also compare anti-*Leishmania*-seronegative dogs with confirmed viral, bacterial or other parasitic infections, non-infectious inflammatory conditions, and conditions without evidence of inflammation. Such comparisons could clarify shared and disease-associated electrophoretic patterns and improve interpretation of future diagnostic datasets.

## 5. Conclusions

In this large retrospective cohort, increasing anti-*Leishmania* antibody titres, as reflected by higher ELISA Rz categories, were associated with a coherent and progressive serum protein electrophoretic phenotype. This phenotype was dominated by gamma-globulin expansion, accompanied by a reciprocal decline in albumin-related ratios. Among the electrophoretic variables evaluated, gamma-globulin concentration showed the strongest association with ELISA Rz category and provided moderate single-marker discrimination of seropositivity, whereas beta-globulin concentration showed a weaker ordinal association. These findings support serum protein capillary electrophoresis as a means of characterising an integrated clinicopathological phenotype associated with anti-*Leishmania* serological intensity in dogs tested for CanL. Quantitative serology remains central to CanL diagnosis, and high titres may confirm disease in a compatible clinical or clinicopathological setting. The proteinogram should not be interpreted as a stand-alone diagnostic test, nor as a direct measure of clinical severity. Rather, it provides a quantitative and visual framework that may refine interpretation when integrated with antibody titre, clinical findings, renal assessment, urinalysis and disease-specific diagnostic testing.

## Figures and Tables

**Table 1:** Proportion of anti-*Leishmania*-seropositive dogs according to age group.

	Anti-*Leishmania*-Seropositive Dogs			Total
Age Groups	*n*	%	95% CI	*n*
<1 year	24	23.8	16.5–32.9	101
1 to <2 years	79	35.4	29.4–41.9	223
2 to <6 years	1037	60.6	58.3–62.9	1710
6 to <11 years	1154	54.6	52.5–56.7	2114
≥11 years	311	43.6	40.0–47.3	713
Total	2605	53.6%	52.2–55.0	4861

Percentages refer to the proportion of seropositive dogs within each age group; 95% confidence intervals (CIs) were calculated using the Wilson method. Age-group association: χ^2^ = 129.27, *df* = 4, *p* < 0.001; Cramer’s *V* = 0.16.

**Table 2:** Serum protein electrophoretic variables in seronegative and seropositive dogs.

Variable	Seronegative Median (IQR)	Seropositive Median (IQR)	*p*	Effect Size (*r*)
Age, years	7.0 (4.0–10.0)	6.0 (4.0–9.0)	0.012	0.045
Total protein, g/dL	6.7 (6.1–7.3)	7.3 (6.6–8.4)	<0.001	0.331
Albumin, %	50.7 (42.5–56.5)	40.1 (30.0–50.2)	<0.001	0.363
Albumin, g/dL	3.4 (2.9–3.7)	3.0 (2.4–3.5)	<0.001	0.220
α1-globulins, %	4.4 (3.3–5.5)	4.0 (2.7–5.1)	<0.001	0.116
α1-globulins, g/dL	0.3 (0.2–0.4)	0.3 (0.2–0.4)	0.483	0.010
α2-globulins, %	11.6 (8.7–14.7)	10.6 (8.4–13.4)	<0.001	0.097
α2-globulins, g/dL	0.76 (0.6–1.0)	0.80 (0.6–1.0)	<0.001	0.047
β-globulins, %	18.9 (15.5–23.3)	19.0 (15.3–23.3)	0.499	0.009
β-globulins, g/dL	1.2 (1.0–1.6)	1.4 (1.1–1.8)	<0.001	0.134
γ-globulins, %	12.0 (8.90–16.7)	21.6 (14.3–33.8)	<0.001	0.461
γ-globulins, g/dL	0.8 (0.58–1.2)	1.5 (1.0–2.7)	<0.001	0.462
Albumin-to-globulin ratio	1.0 (0.7–1.3)	0.7 (0.4–1.0)	<0.001	0.363
Albumin-to-γ-globulin ratio	4.2 (2.6–6.1)	1.9 (0.9–3.4)	<0.001	0.453

IQR, interquartile range.

**Table 3:** Concentrations of γ-globulin according to anti-*Leishmania* serological category.

Serological Category	*n*	Median γ-Globulins, g/dL	95% CI for Median, g/dL	Dunn’s–Bonferroni
Negative	2454	0.79	0.77–0.82	a
Inconclusive	180	0.88	0.79–0.98	a, b
Low positive	368	0.95	0.87–1.02	b
Positive/high positive	1229	1.25	1.19–1.32	c
Very high positive	1313	2.12	2.02–2.24	d
Total	5544	1.07	1.04–1.09	—

CI refer to the median γ-globulin concentration and were calculated using a non-parametric order-statistic approach. Kruskal–Wallis test: χ^2^ = 1553.91, *df* = 4, *p* < 0.001. Serological categories not sharing the same letter differed significantly in Dunn’s–Bonferroni post hoc comparisons. Negative versus inconclusive dogs did not differ significantly after adjustment (adjusted *p* = 0.552), and inconclusive versus low-positive dogs also did not differ significantly after adjustment (adjusted *p* = 1.000). All other pairwise comparisons had adjusted *p* < 0.001.

**Table 4:** Ordinal association between anti-*Leishmania* serological category and selected serum protein electrophoretic variables.

Variable	Spearman’s *ρ*	Kruskal–Wallis ε^2^
γ-globulin concentration (g/dL)	0.522	0.280
γ-globulin fraction (%)	0.521	0.279
Albumin-to-γ-globulin ratio	−0.513	0.272
Albumin fraction (%)	−0.417	0.182
Albumin-to-globulin ratio	−0.416	0.181
Total protein concentration	0.355	0.130
Albumin concentration (g/dL)	−0.258	0.072
β-globulin concentration (g/dL)	0.134	0.018

Spearman’s *ρ* was used to assess the ordinal association between increasing anti-*Leishmania* serological category and each electrophoretic variable. Kruskal–Wallis ε^2^ was retained to describe the magnitude of the overall between-category effect. All Spearman and Kruskal–Wallis tests shown in this table had *p* < 0.001. *ρ*, Spearman’s rank correlation coefficient; ε^2^, epsilon-squared effect size for the Kruskal–Wallis test.

**Table 5:** AUC and respective 95% CI for all electrophoretic variables evaluated.

Variable	AUC	95% CI	Direction
γ-globulin concentration (g/dL)	0.768	0.755–0.780	↑
γ-globulin fraction (%)	0.767	0.754–0.780	↑
Albumin-to-γ-globulin ratio	0.762	0.750–0.775	↓
Albumin fraction (%)	0.711	0.697–0.724	↓
Albumin-to-globulin ratio	0.710	0.696–0.724	↓
Total protein concentration	0.680	0.666–0.694	↑
Albumin concentration (g/dL)	0.627	0.613–0.642	↓
β-globulin concentration (g/dL)	0.578	0.562–0.593	↑

**Table 6:** Pooled study-specific 95% reference intervals for serum protein capillary electrophoretic variables in 254 clinically healthy anti-*Leishmania*-negative dogs aged ≥ 1 to <4 years sampled during 2013–2026.

Variable	Median	IQR	Lower Reference Limit (90% CI)	Upper Reference Limit (90% CI)
Total protein, g/dL	6.40	6.10–6.70	5.6 (5.50–5.60)	7.6 (7.30–7.70)
Albumin, %	55.20	51.40–59.20	43.2 (42.20–44.70)	61.9 (61.50–62.10)
Albumin, g/dL	3.54	3.24–3.78	2.81 (2.79–2.88)	4.14 (4.06–4.17)
α1-globulins, %	4.60	3.50–5.60	0.8 (0.50–1.20)	8.0 (7.20–8.90)
α1-globulins, g/dL	0.29	0.23–0.37	0.05 (0.03–0.07)	0.50 (0.46–0.53)
α2-globulins, %	11.00	8.50–13.60	5.4 (5.30–6.00)	17.3 (16.40–18.40)
α2-globulins, g/dL	0.68	0.55–0.88	0.36 (0.30–0.39)	1.09 (1.04–1.12)
β-globulins, %	18.30	15.60–21.70	9.8 (8.30–10.70)	29.4 (28.30–30.20)
β-globulins, g/dL	1.18	0.98–1.43	0.56 (0.49–0.63)	2.09 (1.96–2.18)
γ-globulins, %	10.30	8.50–13.10	4.9 (4.30–5.80)	19.5 (17.80–21.90)
γ-globulins, g/dL	0.67	0.53–0.85	0.30 (0.26–0.35)	1.26 (1.20–1.36)
A/G	1.23	1.06–1.45	0.76 (0.73–0.81)	1.62 (1.60–1.64)
Albumin-to-γ-globulin ratio	5.28	4.03–6.82	2.53 (2.26–2.73)	11.41 (9.98–14.42)

Data are presented as median, IQR, and non-parametric central 95% reference limits with 90% CI.

## Data Availability

The data presented in this study are available from the corresponding author upon reasonable request. Restrictions apply because the dataset contains anonymised veterinary diagnostic records derived from clinical submissions.

## Abbreviations

The following abbreviations are used in this manuscript:

A/G	albumin-to-globulin ratio
ALB	albumin
ASVCP	American Society for Veterinary Clinical Pathology
AUC	area under the receiver operating characteristic curve
CanL	canine leishmaniosis
CI	confidence interval
CLSI	Clinical and Laboratory Standards Institute
CZE	capillary zone electrophoresis
ELISA	enzyme-linked immunosorbent assay
IgG	immunoglobulin G
IQR	interquartile range
LR+	positive likelihood ratio
LR−	negative likelihood ratio
OR	odds ratio
ROC	receiver operating characteristic
Rz	ELISA ratio
SPE	serum protein electrophoresis
TP	total protein
